# Multivitamins/multiminerals in Switzerland: not as good as it seems

**DOI:** 10.1186/1475-2891-13-24

**Published:** 2014-03-24

**Authors:** Nadège Droz, Pedro Marques-Vidal

**Affiliations:** 1 CHUV, Lausanne, Switzerland and Institute of Social and Preventive Medicine (IUMSP), Lausanne University Hospital, Biopole 2, Route de la Corniche 10, Lausanne CH-1010, Switzerland

**Keywords:** Trace elements, Vitamins, Switzerland, Vitamin supplements

## Abstract

**Background:**

Multivitamin/multimineral (MVM) supplements are commonly consumed by the general population, but little is known regarding their composition and compliance with local regulations. We assessed the composition and compliance with regulations [no indication in the label of vitamin/minerals amounting <15% of the acceptable daily intake (ADI)] of MVM available in Switzerland.

**Methods:**

The composition of vitamin/minerals supplements was obtained from the Swiss drug compendium, the Internet, pharmacies, parapharmacies and supermarkets. MVM was defined as the presence of at least 5 vitamins and/or minerals.

**Results:**

Of the 254 vitamin/mineral supplements collected, 95 (37%) were considered as MVM. The most frequent vitamins were B_6_ (73.7%), C (71.6%), B_2_ (69.5%) and B_1_ (67.4%); the least frequent were K (17.9%), biotin (51.6%), pantothene (55.8%) and E (56.8%). Approximately half of MVMs provided >150% of the ADI for vitamins. The most frequent minerals were zinc (66.3%), calcium (55.8%), magnesium (54.7%) and copper (48.4%), and the least frequent were fluoride (3.2%), phosphorous (17.9%), chrome (22.1%) and iodine (25%). More than two thirds of MVMs provided between 50 and 150% of the ADI for minerals, and few MVMs provided >150% of the ADI. While few MVMs provided <15% of the ADI for vitamins, a considerable fraction did so for minerals (32.7% for magnesium, 26.1% for copper and 22.6% for calcium).

**Conclusion:**

There is a great variability regarding the composition of MVMs available in Switzerland. Several MVM do not comply with Swiss regulations, which calls for monitoring and corrective measures.

## Introduction

Consumption of multivitamin/mineral (MVM) supplements is popular in the general population [1,2]. In Switzerland, 16.8% of the population aged between 35 and 75 consume MVM [1]. In the European Union (EU), MVM represent half of the EU food supplement market, which has been estimated at around five billion Euros in 2005 [3] and over six billion Euros in 2012 [4]. MVM can contain a variable number of vitamins and minerals, the characteristics and amounts ranges of which are regulated [5,6]. Nevertheless, this regulation is relatively supple, as no definition of MVM is currently available, and the amounts of vitamins or minerals allowed per unit (pill, capsule…) can vary considerably. This leads to a large variety of MVM supplements available, which differ in the number and amount of vitamins and minerals included. Hence, comparisons between countries regarding the prevalence of MVM consumers or the amount of vitamins and minerals brought by MVM are difficult to perform. Further, study participants and even nonnutritionist interviewers have been shown to incorrectly identify or record MVMs, thus making between-study comparisons difficult [7]. Similarly, shifting from one MVM supplement to another might lead to changes in vitamin and mineral intake, with possible consequences for health. Indeed, it has been suggested that the effect of vitamin supplementation might differ according to the status of other vitamins [8,9], although other studies failed to show any effect of MVM on mortality (for a review, see [10]).

In this study, we aimed at characterizing the composition and compliance with Swiss legislation of the MVM available in Switzerland.

## Methods

The composition of vitamin/mineral supplements available in Switzerland was obtained from several sources: the Swiss drug compendium, the Internet, pharmacies, parapharmacies and supermarkets. Whenever necessary, further information was gathered by contacting the producer. The geographical search area was restricted to the Vaud canton, but all information in French, German or Italian (official languages in Switzerland) was collected. The following initial inclusion criteria were used: 1) containing at least one vitamin/mineral; 2) taken orally and 3) amount of vitamin/mineral indicated.

### Amount of vitamins and minerals provided by the MVMs

For each vitamin/mineral supplement retained, the composition and the maximal recommended posology (if available) were collected. When the recommended posology was missing, the value of one unit/day was used. The percentage of the acceptable daily intake (“apports journaliers admissibles”) provided by the maximum posology was also calculated and categorized as follows: <15%, 15-150% and >150%. The ADIs were derived from the Swiss legislation [5]. According to the Swiss legislation, if a MVM contains a vitamin or mineral in amounts lower than 15% or the ADI, this vitamin (mineral) should not be indicated on the label [5].

### Statistical analysis

Statistical analyses were conducted using Stata v.12 (Stata corp, College Station, TX, USA). Descriptive results were presented as number (percentage) or median [interquartile range]. As there is no common definition for a multivitamin preparation, two analyses were performed: the first considered all supplements with at least 5 vitamins and/or minerals, and the second included only supplements with at least 10 vitamins and/or minerals.

## Results

Of the 254 vitamin/mineral supplements initially retained, 95 (37%) had at least 5 components and were considered as MVM. Their composition is summarized in Table 1. The most frequent vitamins were B_6_, C, B_2_ and B_1_, while vitamins K and A were present in less than half of the MVMs. The most frequent minerals were zinc, calcium, magnesium and copper, while fluoride, phosphorous, potassium and chrome were present in less than one quarter of the MVMs.

**Table 1:** Presence of, and percentage of the acceptable daily intake provided by different vitamins and minerals in multivitamin-multimineral supplements available in Switzerland containing at least 5 components

	**% of MVM containing**	**Amount§ Median [IQR]**	**% ADI§ Median [IQR]**	**<15% ADI**	**15-150% ADI**	**>150% ADI**
**Vitamins**						
Vitamin A (IE)	34 (35.8)	2667 [1047–3500]	100 [32.5 - 131.2]	4 (11.8)	30 (88.2)	-
Vitamin B_1_ (mg)	64 (67.4)	2.3 [1.4 - 4.5]	205 [127–409]	-	30 (46.9)	34 (53.1)
Vitamin B_12_ (μg)	58 (61.0)	4.5 [2.5 - 10]	180 [100–400]	-	26 (44.8)	32 (55.2)
Vitamin B_2_ (mg)	66 (69.5)	2.2 [1.6 - 4.8]	157 [114–343]	-	33 (50.0)	33 (50.0)
Vitamin B_6_ (mg)	70 (73.7)	3 [2–6]	214 [143–429]	-	27 (38.6)	43 (61.4)
Vitamin C (mg)	68 (71.6)	100 [60–240]	125 [75–300]	2 (2.9)	37 (54.3)	29 (42.7)
Vitamin D (μg)	57 (60.0)	10 [5–15]	200 [100–300]	-	25 (43.9)	32 (56.1)
Vitamin E (mg)	54 (56.8)	17.5 [10–50]	146 [83–417]	-	27 (50.0)	27 (50.0)
Vitamin K (μg)	17 (17.9)	50 [27.5 - 100]	67 [37–133]	-	14 (82.3)	3 (17.7)
Niacin (mg)	61 (64.2)	20 [17–47]	125 [106–294]	-	37 (60.7)	24 (39.3)
Pantothenic acid (mg)	53 (55.8)	16.2 [6–23.5]	269 [100–392]	-	18 (34.0)	35 (66.0)
Folic acid (μg)	56 (59.0)	400 [200–600]	200 [100–300]	1 (1.8)	19 (33.9)	36 (64.3)
Biotin (μg)	49 (51.6)	150 [47.5 - 200]	300 [95–400]	1 (2.0)	20 (40.8)	28 (57.2)
**Minerals**						
Calcium (mg)	53 (55.8)	200 [122.5 - 500]	25 [15–63]	12 (22.6)	37 (69.8)	4 (7.6)
Magnesium (mg)	52 (54.7)	100 [50–187.5]	27 [13–50]	17 (32.7)	33 (63.5)	2 (3.9)
Iron (mg)	39 (41.1)	10 [5.6 - 14]	71 [40–100]	4 (10.3)	30 (76.9)	5 (12.8)
Copper (μg)	46 (48.4)	950 [115–1000]	95 [12–100]	12 (26.1)	27 (58.7)	7 (15.2)
Iodine (μg)	23 (24.2)	150 [75–150]	100 [50–100]	1 (4.3)	20 (87.0)	2 (8.7)
Zinc (mg)	63 (66.3)	10 [7.5 - 15]	100 [75–150]	3 (4.8)	38 (60.3)	22 (34.9)
Manganese (mg)	45 (47.4)	2.0 [1.0 - 3.3]	100 [50–165]	3 (6.7)	29 (64.4)	13 (28.9)
Selenium (μg)	38 (40.0)	50 [27.3 - 70]	91 [50–127]	1 (2.6)	28 (73.7)	9 (23.7)
Chrome (μg)	21 (22.1)	35 [25–50]	88 [63–125]	-	18 (85.7)	3 (14.3)
Molybdenum (μg)	22 (23.2)	50 [45–100]	100 [90–200]	-	15 (68.2)	7 (31.8)
Fluoride (mg)	3 (3.2)	1.5 [0.4 - 3.5]				
Potassium (mg)	21 (22.1)	80 [28–190]	4 [1–10]	16 (76.2)	4 (19.1)	1 (4.8)
Phosphorous (mg)	17 (17.9)	125 [20–142]	18 [3–20]	8 (47.1)	9 (52.9)	-

For components containing at least 5 vitamins or minerals (N=95). IQR, interquartile range; MVM, multivitamin/multimineral; ADI, acceptable daily intake according to the Swiss legislation. Results are expressed as number of products (percentage) or as median [interquartile range]. ^§^for maximum recommended posology; when the posology was not available, a value of 1unit/day was considered. No ADI available for fluoride.

### Amount of vitamins and minerals provided by the MVMs

When the maximum recommended posology was considered, more than half of the MVMs provided intakes >150% of the ADI for all vitamins, excepting niacin and vitamin K (Table 1). Most of the resulting intakes for minerals were between 15% and 150% of the ADI, although higher percentages of MVMs below 15% of ADI were noted for phosphorous, magnesium, copper and calcium (Table 1).

Restricting the analysis to MVM with at least 10 ingredients decreased the number of supplements to 46 (18%) and led to a slight decrease of the intakes representing more than 150% of the ADI for vitamins, while no considerable change was found for minerals (Table 2).

**Table 2:** Presence of, and percentage of the acceptable daily intake provided by different vitamins and minerals in multivitamin-multimineral supplements available in Switzerland containing at least 10 components

	**% of MVM containing**	**Amount§ Median [IQR]**	**% ADI§ Median [IQR]**	**<15% ADI**	**15-150% ADI**	**>150% ADI**
**Vitamins**						
Vitamin A (IE)	29 (63.0)	2667 [1739–3550]	100 [50–131]	1 (3.5)	28 (96.6)	-
Vitamin B_1_ (mg)	44 (95.7)	1.5 [1.4 - 4.2]	136 [127–377]	-	27 (61.4)	17 (38.6)
Vitamin B_12_ (μg)	42 (91.3)	3.5 [2.0 - 8.1]	140 [78–325]	-	21 (50.0)	21 (50.0)
Vitamin B_2_ (mg)	44 (95.7)	1.7 [1.5 - 4.1]	121 [104–293]	-	28 (63.6)	16 (36.4)
Vitamin B_6_ (mg)	46 (100.0)	2.6 [2.0 - 5.2]	186 [143–368]	-	22 (47.8)	24 (52.2)
Vitamin C (mg)	44 (95.7)	90 [60–180]	112.5 [75–225]	-	28 (63.6)	16 (36.4)
Vitamin D (μg)	39 (84.8)	5.5 [5.0 - 12.5]	110 [100–250]	-	20 (51.3)	19 (48.7)
Vitamin E (mg)	41 (89.1)	15 [10.0 - 36.7]	125 [83–305]	-	22 (53.7)	19 (46.3)
Vitamin K (μg)	15 (32.6)	50 [25–75]	67 [33–100]	-	14 (93.3)	1 (6.7)
Niacin (mg)	44 (95.7)	18 [16–30]	113 [100–188]	-	31 (70.5)	13 (29.6)
Pantothenic acid (mg)	38 (82.6)	10 [6–20]	167 [100–333]	-	16 (42.1)	22 (57.9)
Folic acid (μg)	41 (89.1)	400 [200–407]	200 [100–204]	-	14 (34.2)	27 (65.9)
Biotin (μg)	39 (84.8)	75 [40–200]	150 [80–400]	-	18 (46.2)	21 (53.9)
**Minerals**						
Calcium (mg)	33 (71.4)	180.6 [100–239]	23 [13–30]	9 (27.3)	24 (72.7)	-
Magnesium (mg)	35 (76.1)	60 [40–100]	16 [11–27]	17 (48.6)	18 (51.4)	-
Iron (mg)	30 (65.2)	10 [7–15]	71 [50–107]	2 (6.7)	24 (80.0)	4 (13.3)
Copper (μg)	30 (65.2)	900 [367.5 - 1000]	90 [37–100]	7 (23.3)	20 (66.7)	3 (10.0)
Iodine (μg)	22 (47.8)	150 [71.3 - 150]	100 [48–100]	1 (4.6)	19 (86.4)	2 (9.1)
Zinc (mg)	41 (89.1)	10 [6.8 - 15]	100 [68–150]	3 (7.3)	27 (65.9)	11 (26.8)
Manganese (mg)	31 (67.4)	2 [1–3]	100 [50–150]	2 (6.5)	21 (67.7)	8 (25.8)
Selenium (μg)	25 (54.4)	50 [26.5 - 57.5]	91 [48–105]	1 (4.0)	19 (76.0)	5 (20.0)
Chrome (μg)	19 (41.3)	35 [25–50]	88 [63–125]	-	16 (84.2)	3 (15.8)
Molybdenum (μg)	21 (45.7)	50 [45–100]	100 [90–200]	-	14 (66.7)	7 (33.3)
Fluoride (mg)	3 (6.5)	1.5 [0.4 - 3.5]				
Potassium (mg)	12 (26.1)	80 [22–80]	4 [1–4]	11 (91.7)	1 (8.3)	-
Phosphorous (mg)	15 (32.6)	50 [20–133.3]	7 [3–19]	8 (53.3)	7 (46.7)	-

For components containing at least 10 vitamins or minerals (N=46). IQR, interquartile range; MVM, multivitamin/multimineral; ADI, acceptable daily intake according to the Swiss legislation. Results are expressed as number of products (percentage) or as median [interquartile range]. ^§^for maximum recommended posology; when the posology was not available, a value of 1/day was considered.

## Discussion

To our knowledge, this is the first study in Switzerland and in Europe to assess the composition and compliance to regulations of MVM. Our results show that the composition of MVMs varies considerably according to the definition applied. For instance, if a loose definition is used (at least 5 vitamins or minerals), a sizable fraction of the MVMs lacks specific vitamins or minerals; using a more restrictive criterion (at least 10 vitamins or minerals) leads to products that contain mostly all vitamins and minerals. Hence, we propose that MVMs be defined as components that contain at least 10 different vitamins or minerals, as a first step for standardization and comparison between studies. Still, such standardization will be very difficult to achieve, as current national regulations do not impose a fixed number of components or a specific amount for each component in MVMs. Further, even within a single country, there are wide differences in the definition of MVM, ranging from at least one vitamin and one mineral plus other ingredients to 3 vitamins with or without minerals [7]. Still, and whenever possible, investigators should indicate which definition of MVM they use in their study.

### Amount of vitamins and minerals provided by the MVMs

Slightly more than half of all MVMs provided vitamins in amounts exceeding 150% of the ADI, while the percentage of MVMs providing minerals exceeding the 150% threshold was considerably lower. Interestingly, MVMs with at least 10 vitamins and minerals appeared to contain lower doses than MVMs with at least 5 vitamins or minerals. The reasons for such a discrepancy are unclear and can only be speculated. Hydrosoluble vitamins consumed in excess can be excreted via the kidney, while no such mechanism is available for some minerals; hence, the amount of minerals might be reduced for safety reasons. Even though, water-soluble vitamins in excess can have adverse health effects. For instance, ascorbic acid supplements have been associated with a 2-fold increase in the incidence of kidney stones [11] and can also lead to oxalosis in subjects with reduced renal function [12]. Finally, MVMs with a large number of vitamins and minerals might be more expensive, thus tempting the manufacturers to reduce the amount of vitamins and minerals present per unit. Still, further research is needed to clarify this issue.

The main iodine source in Switzerland is iodized table salt, which has proved a successful preventive measure against goiter and iodine deficiency [13,14]. Still, recommendations towards lower salt intake [15] and the use of fashionable, exotic (and non-iodized) salt by the Swiss population might actually lead to an increase in iodine deficiency [16]. Somewhat unexpectedly, the number of MVMs containing iodine was rather low, while it could be of benefit for a fraction of the Swiss population. Indeed, MVM consumers tend to pursue a healthier dietary pattern and to be more respondent to dietary recommendations, including salt reduction. Hence, in these subjects, the lower iodine intake due to lower salt intake could be compensated by MVMs.

Vitamin K, a major determinant of blood coagulation, was present in one out of six MVMs and in one out of three if the threshold of at least 10 vitamins or minerals was used. Importantly, vitamin K was mainly indicated for prevention against arthritis, and no information regarding its effect on coagulation or its interaction with oral anticoagulants was found. This lack of information can have serious health consequences, namely in MVMs whose content of vitamin K exceeds 150% of the ADI. For instance, elderly people presenting with arthritis (and some of which on oral anticoagulants) might be tempted to consume MVM containing vitamin K, thus modifying their coagulation status and ultimately their health [17]. Although most patients on oral anticoagulants are aware of the need to check their sources of vitamin K, still many believe that MVM are equivalent and might compromise their treatment when shifting from one brand of MVM to another. Finally, very few MVMs contained fluoride, probably in order to prevent any adverse effect of excessive consumption [18].

### Compliance with national regulations

Several MVMs indicated in their label vitamins or minerals in amounts lower than 15% of the ADI. These MVM do not comply with Swiss regulations, which indicate that if a MVM contains a vitamin (mineral) in amounts lower than 15% or the ADI, this vitamin (mineral) should not be stated on the label [5]. Inadequate labeling concerned mainly calcium, magnesium and copper, while very few vitamins had values below the 15% level. Marketing issues might explain this finding, as MVMs with calcium and magnesium indicated in the label might be more appealing to the elderly (as prevention for osteoporosis) and to subjects with anxiety [19], respectively. Still, further studies are needed to better assess this point.

### Study strengths and limitations

Our study provides important information for epidemiologic studies investigating the association between MVM and chronic diseases and also for communicating results from such studies to the general public. The considerable variation in doses and included vitamins and minerals in MVM observed makes it hard to adequately measure exposure to MVMs and to compare studies assessing MVM use.

This study has also several limitations that should be accounted for. First, the collection of vitamin and mineral supplements was limited to the canton of Vaud, and it is possible that other products are available in other cantons. Still, all vitamin supplements from the Swiss drug compendium and from national producers and sellers were collected, which might limit the number of supplements missed. Second, there is no standard definition of MVM, so the results and conclusions might change if other definitions are proposed. Thus, a minimal standardization of the definition of MVM is needed, which will allow comparing between studies. Third, it was not possible to validate the vitamin and mineral content of the MVM, due to the lack of funding. It will be of interest that the Swiss agency for therapeutic products performs such controls, at least in a limited sample of MVMs. Fourth, some MVMs had no posology indicated. Thus, a maximal posology of one unit per day was considered, as it was the most frequently recommended.

## Conclusion

Multivitamin/multimineral supplements available in Switzerland vary considerably regarding their composition and amount of vitamins and minerals. Several MVM do not comply with the Swiss regulations, which calls for monitoring and corrective measures.

## Competing interests

The authors report no competing interests.

## Authors’ contributions

ND collected the data and wrote part of the manuscript. PMV analyzed the data and revised the manuscript. PMV had full access to the data and is the guarantor of the study. ND and PMV have read and approved the manuscript. Both authors read and approved the final manuscript.
